# Dr Robin Biellik (1949 — 2024)

**DOI:** 10.11604/pamj.supp.2025.51.1.47434

**Published:** 2025-06-11

**Authors:** Jean-Marie Okwo-Bele

**Affiliations:** 1 Immunisation consultant, public health expert, former Director of the Department of Immunization, Vaccines and Biologicals of the World Health Organization (WHO) (2004-2017), Geneva, Switzerland

**Keywords:** Robin Biellik, immunization, measles elimination, obituary, expanded program on immunization

**Figure 1:**
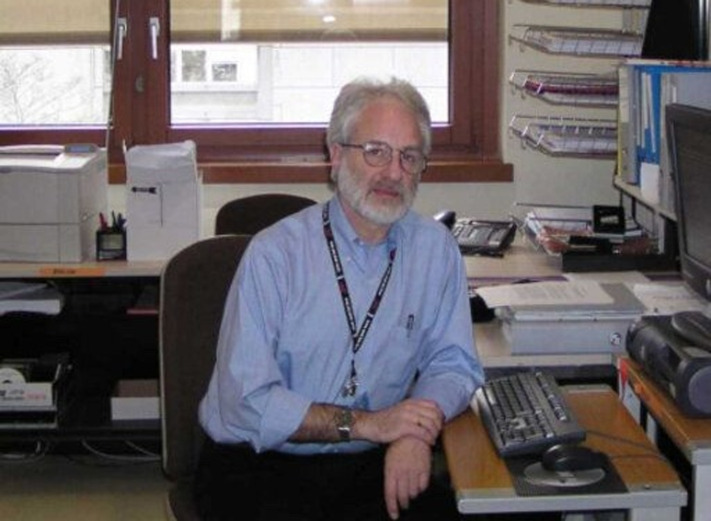
Dr Robin Biellik

It is with profound sorrow that we announce the passing of Dr. Robin Biellik (Figure 1) on the morning of Saturday, December 7, 2024, following a courageous battle with cancer. He was 75 years old. Robin faced his illness with remarkable grace and resilience, enduring tremendous pain while remaining steadfastly committed to his work and loved ones. In a touching message during his hospital stay, he wrote to me, “The leukemia has not let me go, but I´ve had all the brain radiotherapy anyone can have. We´ll see how long this keeps me going. I would be delighted to see you, but rather not in bed in a hospital gown. I should be settled back at home...”. This sentiment exemplified Robin’s unwavering spirit, as he sought to engage with others on his own terms, prioritizing connection even in difficult times.

Robin passed away peacefully, attended by his beloved wife. He was a stalwart of the Expanded Program on Immunization (EPI), serving as a living testament to the history and evolution of public health initiatives. His profound commitment to childhood immunization and evidence-based public health made him a champion for the cause, significantly demonstrating the impact of herd immunity, particularly against measles. As an institutional memory of the program, Robin frequently reminded colleagues that ideas deemed new had often been proposed 10 to 15 years earlier, prompting teams to innovate and adapt to advance these initiatives.

His sharp intellect was complemented by a unique ability to challenge ideas constructively. Robin presented criticisms that, while objective, drove his colleagues to strengthen their assertions and consider alternative viewpoints. He was unafraid to voice his opinions, even when they deviated from the majority perspective, ensuring that groupthink never took root in discussions.

Beyond his professional contributions, Robin had a deep passion for photography. In his final days, he was dedicated to launching an exhibition titled “Flowers, Lace, and Promises,” showcasing the diverse wedding settings, attire, and features he encountered during his travels around the globe. This exhibition, held at an art gallery in December to February, exemplified his ability to find beauty in life´s fleeting moments.

Robin was also known for his warmth and kindness, nurturing friendships with colleagues that spanned decades. In response to the many well-wishes he received on his 75^th^ birthday, he expressed his gratitude, stating, “I didn’t see it coming, and it is representative of all regions and aspects of my professional career. Acknowledgment and recognition are priceless.” He concluded his message with a heartfelt, “An eternal thank you to each wonderful colleague for our time together.”

More than a mentor, Robin was an inspiration and guiding light to many of us in the early decades of EPI and polio eradication in Africa. His loss is not only a significant blow to the childhood immunization advocacy community but also to all who had the privilege of calling him a friend. Robin´s contributions to global health and his legacy of kindness and integrity will resonate for years to come.

As we mourn the passing of this remarkable individual, our thoughts go out to his wife, Peggy, and all those touched by Robin’s life and work. Though he may be gone, his influence and spirit will forever remain in our hearts and efforts.

Contributions from: Dr Jean-Marie Okwo-Bele (Immunisation consultant, public health expert, former Director of the Department of Immunization, Vaccines and Biologicals of the World Health Organization (WHO) (2004-2017), Geneva, Switzerland.

